# Alcohol Consumption Is a Risk Factor of Surgical Site Infection After Minimally Invasive Surgery: A Secondary Observational Analysis of a Clinical Trial

**DOI:** 10.1002/ags3.70210

**Published:** 2026-03-02

**Authors:** Toshiya Akai, Makoto Takeda, Eisuke Booka, Tomohiro Matsumoto, Masayo Takaoka, Mayu Sakata, Yoshifumi Morita, Hirotoshi Kikuchi, Yoshihiro Hiramatsu, Hiroya Takeuchi

**Affiliations:** ^1^ Department of Surgery Hamamatsu University School of Medicine Hamamatsu Japan; ^2^ Data Ops Center Hamamatsu University School of Medicine Hamamatsu Japan; ^3^ Department of Perioperative Functioning Care and Support Hamamatsu University School of Medicine Hamamatsu Japan

**Keywords:** alcohol consumption, minimally invasive surgery, surgical site infection

## Abstract

**Background:**

Surgical site infection (SSI) is one of the postoperative complications. Risk factors for SSI primarily pertain to laparotomy, particularly within the field of gastroenterological surgery. It has been reported that laparoscopic surgery is associated with a lower incidence of SSI compared to open surgery; however, minimally invasive surgery (MIS) has become increasingly widespread in recent years. Therefore, the aim of this study was to examine the risk factors for SSI specifically in patients undergoing MIS.

**Methods:**

This study is a secondary analysis of 529 patients selected from a previously reported clinical trial that focused on gastroenterological surgery and included 697 participants. The patient undergoing elective MIS were divided into two groups based on the occurrence of SSI, and the risk factors associated with SSI in MIS were examined.

**Results:**

SSI within 30 days after surgery was observed in 62 patients (11.7%). In the univariate analysis, male sex, alcohol consumption, bleeding, and operation time were significantly more frequent in the SSI group (*p* < 0.05). Multivariate analysis was performed including these variables as well as current smoking, immunosuppressive drugs, diabetes, and serum albumin. Only alcohol consumption showed a statistically significant association with SSI (odds ratio: 2.818, 95% confidence interval: 1.510–5.260, *p* = 0.001).

**Conclusion:**

The findings of this study revealed that alcohol consumption is significantly associated with SSI occurrence in patients undergoing MIS for gastroenterological indications. Whether preoperative abstinence from alcohol prevents the occurrence of SSI warrants further investigation.

## Graphical abstract

Alcohol consumption was independently associated with Surgical Site Infection in the gastroenterological Minimally Invasive Surgery. Whether preoperative abstinence from alcohol prevents the occurrence of SSI warrants further investigation.

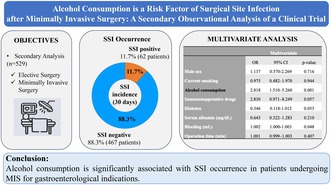

## 1 Introduction

Surgical site infection (SSI) is a postoperative complication influenced by various risk factors, such as age, nutrition, diabetes mellitus, smoking, obesity, immune status, operation time, the use of surgical drains, and surgical procedures [1]. Preventive measures for SSI include skin disinfection and prophylactic antibiotics.

In recent years, minimally invasive surgery (MIS) has become increasingly widespread, and its usefulness has been reported in many cases [2, 3]. MIS decreases the incidence of SSI compared to open surgery [4, 5, 6]. However, the risk factors for SSI have been analyzed only in relation to open surgery. The risk factors for SSI in MIS are unknown; thus, considering the in the era of MIS, it is essential to examine new risk factors for SSIs. Robotic surgery has been identified as a suppressive factor for SSI compared to open or laparoscopic surgery; for example, robotic or laparoscopic surgery for pancreaticoduodenectomy (PD), esophagectomy [7, 8].

Previously, we conducted a randomized controlled study to investigate the usefulness and adverse events of wound irrigation with aqueous povidone‐iodine (PI) solution in gastrointestinal and hepatobiliary‐pancreatic surgeries [9]. As more than 80% of the participants in that study underwent MIS, the current study focused on those scheduled for elective MIS to evaluate the risk factors for SSI. We hypothesized that factors other than those reported as SSI risk factors influence its incidence in MIS. Thus, the aim of this study is to identify the risk factors of SSI in patients scheduled for elective MIS.

## 2 Material and Methods

### 2.1 Study Design

This study is a secondary analysis of our randomized controlled trial, which is registered with the Japan Registry of Clinical Trials. The trial compared the efficacy of using PI for irrigation following normal sterile saline irrigation, in terms of preventing SSI, with normal sterile saline irrigation alone in gastroenterological surgery. In conclusion, PI wound irrigation has been shown to have no additional beneficial effect on the occurrence of surgical site infections [9]. A total of 697 patients were enrolled in the previous trial. Details of the study design, inclusion/exclusion criteria, and efficacy and safety results have been described previously [9]. In brief, the primary outcome was a 30‐day SSI incidence, as defined by the 2017 Centers for Disease Control and Prevention (CDC) SSI guidelines 2017 [10]. Surgical wound classification was defined according to the CDC guideline for prevention of SSI 1999 [11] as follows:

- Class I (Clean): An uninfected operative wound with no signs of inflammation, and where the respiratory, alimentary, genital, or uninfected urinary tract is not involved.
- Class II (Clean‐Contaminated): Operative wounds in which the respiratory, alimentary, genital, or urinary tracts are involved under controlled conditions without unusual contamination. Specifically, operations involving the biliary tract, appendix, vagina, and oropharynx are included in this category, provided no evidence of infection or major break in technique is encountered.
- Class III (Contaminated): Open, fresh, accidental wounds, including operations with major breaks in the sterile technique or gross spillage from the gastrointestinal tract.
- Class IV (Dirty‐Infected): Old traumatic wounds with retained devitalized tissue and those involving existing clinical infection or perforated viscera.

The data analysts, who did not attend the operation and were blinded to the treatment allocation, included a vascular surgeon, two nurses, and a pharmacist. They assessed the wound status for the presence or absence of SSI using electronic medical records, including wound photographs.

The present study was approved by the Clinical Research Review Board of Hamamatsu University School of Medicine (Approval No. 24‐040) and was conducted in accordance with the international ethical principles of the Declaration of Helsinki and the Ethical Guidelines for Medical and Health Research Involving Human Subjects.

Patients were selected after randomization and data analysis based on the subgroup defined before secondary analysis. The definition included patients who underwent elective MIS (529 out of 697), excluding those who underwent open (*n* = 1344) and emergency (*n* = 34) surgery.

### 2.2 Statistical Analysis

Univariate analyses using the *t*‐test, Mann–Whitney test, and Chi‐squared test, and multivariable logistic regression were performed to assess the associations between various factors (*p* < 0.05) and the incidence of 30‐day SSI. Variables included in the multivariate logistic regression model were selected based on clinical relevance and previous literature regarding risk factors for SSI, while considering the limited number of SSI events to avoid overfitting. The model was constructed in consultation with a professional statistician.

The factors included were age, sex, body mass index, current smoking status (defined as patients who smoked daily at the time of their initial visit to our hospital), alcohol consumption, ASA‐PS (American Society of Anesthesiologists‐Physical Status), diabetes mellitus, use of immunosuppressive drugs, type of surgery (upper gastrointestinal, lower gastrointestinal, hepatobiliary‐pancreatic), operation time, blood loss, wound classification, incidence of SSI, and results of preoperative blood tests, including white blood cell count, hemoglobin, platelet count, total bilirubin, aspartate aminotransferase, alanine aminotransferase, albumin, cholinesterase, blood urea nitrogen, creatinine, C‐reactive protein, prothrombin time (%), and activated partial thromboplastin time (%). Alcohol consumption was assessed using self‐reported data at the time of their initial visit. Based on Japanese clinical guidance [12], habitual alcohol consumption was defined as an intake of ≥ 20 g of alcohol per day on an almost daily basis. Patients who had abstained from alcohol for more than 1 year and occasional/social drinkers were categorized into the non‐alcohol group. All statistical analyses were performed using SPSS software (version 30). The plan was developed by the principal investigator and biostatistician before the completion of patient recruitment and data fixation.

## 3 Results

**FIGURE 1:**
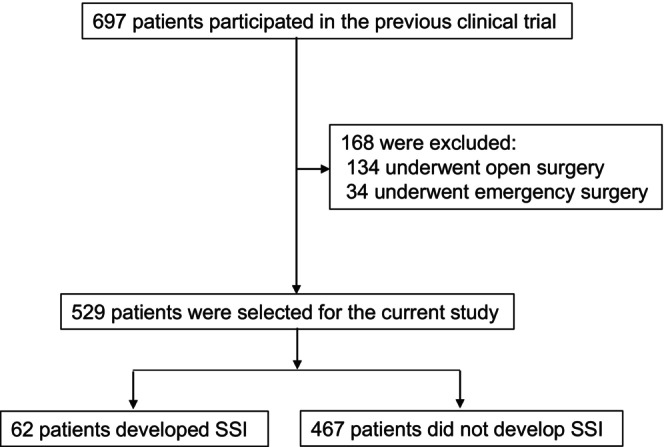
The trial profile.

In the previous trial, 697 patients were eligible for the full analysis set between November 9, 2020, and December 23, 2022, to determine the efficacy of wound irrigation with PI. Among them, 529 patients who met the eligibility criteria (elective surgery and MIS [laparoscopic, thoracic, or robotic surgery]) were selected for the current study (Figure 1). The 134 patients who underwent open surgery and 34 who underwent emergency surgery were excluded.

The patient characteristics were as follows (Table 1): 18.7% (99/529) were smokers, 35.7% (189/529) reported current alcohol use, and 42.9% (227/529) had undergone lower gastrointestinal surgery. The incidence rate of SSI was 11.7% (62/529).

**TABLE 1:** Baseline characteristics of the patients.

Sex: male/female	320 (60.5%)/209 (39.5%)
Age (years), median (IQR)	68.0 (54.0–76.0)
Current smoking status: yes/no	99 (18.7%)/430 (81.3%)
Alcohol consumption: yes/no	189 (35.7%)/340 (64.3%)
ASA‐PS: I/II/III	56 (10.6%)/412 (77.9%)/61 (11.5%)
BMI (kg/m^2^), median (IQR)	22.6 (20.4–25.6)
Diabetes: yes/no	78 (14.7)/451 (85.3%)
Immunosuppressive drugs: yes/no	25 (4.7%)/504 (95.3%)
Surgical site	
Upper gastrointestinal	190 (35.9%)
Lower gastrointestinal	227 (42.9%)
Hepatobiliary pancreatic	112 (21.2%)
Bleeding (mL), median (IQR)	22.0 (6.0–68.5)
Operation time (min), median (IQR)	257 (169–395)
Wound classification; II/III/IV	501 (94.7%)/25 (4.7%)/3 (0.6%)
Intraoperative blood transfusion	59 (11.2%)
Wound irrigation with povidone‐iodine	264 (50%)
SSI: yes/no	62 (11.7%)/467 (88.3%)
Incisional/organ space	29 (46.8%)/33 (53.2%)

*Note:* Continuous variables are expressed as median (interquartile range) due to non‐normal distribution.

Abbreviations: ASA‐PS, American Society of Anesthesiologists‐Physical Status; BMI, body mass index; IQR, interquartile range; SSI, surgical site infection.

The main surgical procedures were esophagectomy, gastrectomy, small bowel resection, appendectomy, colorectal resection, cholecystectomy, hepatectomy, and distal pancreatectomy. However, PD or hepatectomy with biliary reconstruction was not performed because all procedures were done using open techniques. Subgroup analyses were performed according to surgical field (upper gastrointestinal, lower gastrointestinal, and hepatobiliary–pancreatic surgery). These results were presented in Table S1. No statistically significant difference in SSI incidence was observed among the subgroups.

In univariate analysis, SSI occurred significantly more often in males and in patients with a history of alcohol consumption, greater blood loss, and longer operation times (Table 2). Patients with SSI also showed increased creatinine levels (*p* = 0.034; Table S2). Multivariate logistic regression analysis included male sex, current smoking, alcohol consumption, use of immunosuppressive drugs, diabetes, serum albumin, bleeding, and operation time. Although serum creatinine showed statistical significance in the univariate analysis, the median values were within the institutional reference range. Moreover, serum creatinine has not been consistently reported as a risk factor for SSI in previous studies [13]. Therefore, serum creatinine was excluded from multivariate analysis.

**TABLE 2:** Characteristics of all observed SSIs.

	SSI (+) *N* = 62	SSI (−) *N* = 467	*p*
Sex: male/female	46 (74.2%)/16 (25.8%)	274 (58.7%)/193 (41.3%)	0.019
Age (years), median (IQR)	69.0 (57.0–78.2)	68.0 (54.0–75.0)	0.284
Current smoking	13 (21.0%)	86 (18.4%)	0.628
Alcohol consumption	38 (55.1%)	151 (32.3%)	< 0.001
ASA‐PS: I, II/III	57 (91.9%)/5 (8.1%)	411 (88.0%)/56 (12.0%)	0.363
BMI (kg/m^2^), median (IQR)	21.9 (20.5–24.6)	22.8 (20.3–25.6)	0.253
Diabetes	4 (6.5%)	74 (15.8%)	0.050
Immunosuppressive drugs	6 (9.7%)	19 (4.0%)	0.101
Surgery			
Upper gastrointestinal	28 (45.2%)	162 (34.7%)	0.261
Lower gastrointestinal	22 (35.5%)	205 (43.9%)	
Hepatobiliary pancreatic	12 (19.3%)	100 (21.4%)	
Bleeding (mL), median (IQR)	45.5 (10.0–149.5)	20.0 (5.0–63.0)	0.012
Operation time (min), median (IQR)	330.0 (199.5–482.0)	251.0 (166.0–382.0)	0.006
Intraoperative blood transfusion	11 (17.7%)	48 (10.3%)	0.079
Wound classification: II/III, IV	60 (96.8%)/2 (3.2%)	441 (94.4%)/26 (5.6%)	0.761
Wound irrigation with povidone‐iodine	38 (61.3%)	226 (48.4%)	0.056

Abbreviations: ASA‐PS, American Society of Anesthesiologists‐Physical Status; BMI, body mass index; SSI, surgical site infection.

The results identified alcohol consumption (OR = 2.818; 95% CI, 1.510–5.260; *p* = 0.001), as independent risk factors for SSI (Table 3).

**TABLE 3:** Multivariate analysis of factors associated with overall SSI occurrence.

	Univariable			Multivariable		
	OR	95% CI	*p*	OR	95% CI	*p*
Male sex	2.024	1.135–3.676	0.019	1.137	0.570–2.269	0.716
Current smoking	1.175	0.611–2.262	0.628	0.975	0.482–1.970	0.944
Alcohol consumption	3.311	1.919–5.714	< 0.001	2.818	1.510–5.260	0.001
Immunosuppressive drugs	2.526	0.968–6.591	0.101	2.830	0.971–8.249	0.057
Diabetes	0.366	0.129–1.040	0.050	0.346	0.118–1.012	0.053
Serum albumin (mg/dL)			0.138	0.643	0.322–1.283	0.210
Bleeding (mL)			0.012	1.002	1.000–1.003	0.048
Operation time (min)			0.006	1.001	0.999–1.003	0.407

Abbreviations: CI, confidence interval; OR, odds ratio; SSI, surgical site infection.

Incisional SSI occurred more frequently in patients who current smoking, alcohol consumption, had elevated serum aspartate aminotransferase levels, and received PI irrigation in univariate analysis (*p* < 0.05; Table 4, Table S3). Serum aspartate aminotransferase and PI irrigation were not included in multivariate analysis, as they are not recognized SSI risk factors. The multivariate logistic regression analysis for incisional SSI included diabetes, bleeding and operation time in addition to current smoking and alcohol consumption. Alcohol consumption and bleeding were identified as significant risk factors (odds ratio [OR] = 2.397; 95% confidence interval [CI]: 1.076–5.340; *p* = 0.032, OR = 1.002; 95% CI, 1.000–1.005; *p* = 0.027) (Table 5).

**TABLE 4:** Characteristics of incisional SSI occurrence.

	Incisional SSI (+) *N* = 29	Incisional SSI (−) *N* = 500	*p*
Sex: male/female	16 (55.2%)/13 (44.8%)	304 (60.8%)/196 (39.2%)	0.547
Age (years), median (IQR)	69.0 (58.0–79.5)	68.0 (54.0–75.0)	0.317
Current smoking	10 (34.4%)	89 (17.8%)	0.025
Alcohol consumption	17 (58.6%)	172 (34.4%)	0.008
ASA‐PS: I, II/III	27 (93.2%)/2 (6.8%)	441 (88.2%)/59 (11.8%)	0.561
BMI (kg/m^2^), median (IQR)	22.3 (21.0–26.4)	22.6 (20.3–25.5)	0.997
Diabetes	2 (6.8%)	76 (15.2%)	0.288
Immunosuppressive drugs	1 (3.4%)	24 (4.8%)	1.000
Surgical site			
Upper gastrointestinal	10 (33.5%)	180 (36.0%)	0.380
Lower gastrointestinal	10 (33.5%)	217 (43.4%)	
Hepatobiliary pancreatic	9 (31.0%)	103 (20.6%)	
Bleeding (mL), median (IQR)	35.0 (0.5–161.5)	21.0 (7.0–67.0)	0.872
Operation time (min), median (IQR)	286.0 (125.5–422.0)	257.0 (169.0–394.2)	0.957
Intraoperative blood transfusion	5 (17.2%)	54 (10.8%)	0.355
Wound classification: II/III, IV	29 (100%)/0 (0%)	472 (94.4%)/28 (5.6%)	0.390
Wound irrigation with povidone‐iodine	21 (72.4%)	243 (48.6%)	0.013

Abbreviations: ASA‐PS, American Society of Anesthesiologists‐Physical Status; BMI, body mass index; SSI, surgical site infection.

**TABLE 5:** Multivariate analysis of factors associated with incisional SSI occurrence.

	Univariable			Multivariable		
	OR	95% CI	*p*	OR	95% CI	*p*
Current smoking	2.433	1.092–5.405	0.025	2.217	0.964–5.100	0.061
Alcohol consumption	2.702	1.261–5.780	0.008	2.397	1.076–5.340	0.032
Diabetes	0.413	0.096–1.774	0.288	0.383	0.087–1.681	0.204
Bleeding (mL)			0.872	1.002	1.000–1.005	0.027
Operation time (min)			0.957	1.000	0.997–1.002	0.239

Abbreviations: CI, confidence interval; OR, odds ratio; PI, povidone iodine; SSI, surgical site infection.

Organ/space SSI occurred more frequently in males, in patients taking immunosuppressive drugs, those who consumed alcohol, and those with greater blood loss, longer operation times, and elevated serum creatinine levels in univariate analysis (*p* < 0.05; Table 6, Table S4). In the multivariate logistic regression analysis for organ/space SSI, diabetes was added to these factors. The results revealed that the use of immunosuppressive drugs (OR = 7.479; 95% CI, 2.267–24.675; *p* < 0.001) was a significant risk factor (Table 7).

**TABLE 6:** Characteristics of organ/space SSI occurrence.

	Organ/space SSI (+) *N* = 33	Organ/space SSI (−) *N* = 496	*p*
Sex: Male/female	30 (90.9%)/3 (9.1%)	290 (58.5%)/206 (41.5%)	< 0.001
Age (years), median (IQR)	69.0 (53.5–78.0)	68.0 (54.0–75.0)	0.317
Current smoking	3 (9.1%)	96 (19.3%)	0.143
Alcohol consumption	21 (63.6%)	168 (33.8%)	0.001
ASA‐PS: I, II/III	30 (91.9%)/3 (9.1%)	438 (88.4%)/58 (11.6%)	1.000
BMI (kg/m^2^), median (IQR)	21.8 (19.9–23.4)	22.7 (20.4–25.6)	0.067
Diabetes	2 (6.0%)	76 (15.3%)	0.204
Immunosuppressive drugs	5 (15.1%)	20 (4.0%)	0.015
Surgical site			
Upper gastrointestinal	18 (54.5%)	172 (34.7%)	0.046
Lower gastrointestinal	12 (36.4%)	215 (43.3%)	
Hepatobiliary pancreatic	3 (9.1%)	109 (22.0%)	
Bleeding (mL), median (IQR)	60.0 (18.5–156.5)	20.0 (5.0–25.0)	0.001
Operation time (min), median (IQR)	388.0 (251.5–499.5)	252.5 (165.2–384.2)	< 0.001
Intraoperative blood transfusion	6 (18.1%)	53 (10.6%)	0.245
Wound classification: II/III, IV	31 (94.0%)/2 (6.0%)	470 (94.8%)/26 (5.2%)	0.691
Wound irrigation with povidone‐iodine	17 (51.5%)	247 (49.7%)	0.849

Abbreviations: ASA‐PS, American Society of Anesthesiologists‐Physical Status; BMI, body mass index; SSI, surgical site infection.

**TABLE 7:** Multivariate analysis of factors associated with organ/space SSI occurrence.

	Univariate			Multivariate		
	OR	95% CI	*p*	OR	95% CI	*p*
Male sex	7.103	2.139–23.588	< 0.001	4.848	1.300–16.808	0.015
Immunosuppressive drugs	4.255	1.485–12.19	0.015	7.479	2.267–24.675	< 0.001
Alcohol consumption	3.412	1.642–7.092	< 0.001	2.098	0.943–4.666	0.069
Diabetes	0.357	0.084–1.521	0.204	0.340	0.077–1.497	0.154
Bleeding (mL)			0.001	1.001	0.999–1.003	0.520
Operation time (min)			< 0.001	1.003	1.000–1.005	0.047

Abbreviations: CI, confidence interval; OR, odds ratio; SSI, surgical site infection.

## 4 Discussion

The risk factors for SSI after MIS were identified in this secondary analysis comprising patients participating in another clinical trial [9]. The previous trial included more than 80% of patients who underwent MIS, reflecting the increasing prevalence in the gastroenterological field [14]. Therefore, we considered this population suitable for the secondary analysis. The current study is the first to focus on gastroenterological MIS and reports a strong association between alcohol consumption and SSI occurrence.

The risk factors of SSI were reported as follows: ASA‐PS ≥ 3 [15], wound classification ≥ 3 [15], smoking [11], malnutrition [11], prolonged operation time [15], body mass index ≥ 25 kg/m^2^ [16], postoperative hyperglycemia (> 200) [17], intraoperative hypothermia (< 36°C) [17], emergency surgery [15], use of steroids or immunosuppressive drugs [18, 19], and preoperative radiotherapy for surgical fields [20, 21]. However, alcohol consumption was not a definitive risk factor for SSI.

In open surgery, surgical invasiveness may be a dominant determinant of postoperative SSI, and the impact of patient‐related factors may be relatively masked. In contrast, MIS is associated with less tissue damage, smaller incision wounds, and reduced inflammatory responses, resulting in a lower baseline SSI incidence [5]. Therefore, in the MIS era, patient‐related factors such as alcohol consumption may become more detectable as independent risk factors for SSI.

Care bundles have been recommended as measures for preventing SSI, allowing each institution to implement various strategies tailored to its specific needs. For example, measures such as shortening preoperative hospitalization, improving malnutrition, abstaining from smoking, refraining from shaving with razors, administering antibiotic prophylaxis, exchanging surgical instruments before wound closure, using antibacterial absorbent thread for wound closure, applying dermal sutures with absorbable materials, controlling blood sugar during the perioperative period, providing intraoperative warming, and dressing wounds were implemented in the real world to prevent SSIs [1, 10]. Additionally, our institution has implemented various measures for SSI, including improving nutrition, prehabilitation [22], cessation of smoking (at least 4 weeks before surgery), and appropriate antibiotic prophylaxis. The results of our study are based on the implementation of preventive measures for SSI. Although malnutrition is a risk factor for SSI, serum albumin—one of the nutritional indicators—did not differ between the two groups in the current study. Given the various measures used, alcohol consumption was found to be most strongly associated with overall SSI. Overall, alcohol consumption was most strongly associated with SSI. The lack of preventive measures for alcohol consumption may have contributed to SSI occurrence in this study.

The factors affecting the physical effects of alcohol consumption included genetic factors, sex, age, body weight, and lifestyle diseases [23], some of which were identified as risk factors for SSI. This study showed that alcohol consumption was a strong risk factor for SSI. The American National Surgery Quality Improvement Program defined alcohol consumption as a risk factor for surgical morbidity when it exceeds 2 U (24 g) /day (1 U of alcohol = 12 g of alcohol), regardless of gender [24, 25]. In Japan, moderate alcohol consumption is generally defined as up to 20 g per day (lowest mortality rate) regardless of sex [12]. Angus et al. [26]. Reported that preoperative alcohol consumption increases mortality within 30 days, anastomotic leakage, and SSI. In this study, alcohol consumption in this study was defined as ≧ 14 units per week (≧ 16 g/day) or more, which aligns with the European recommended alcohol intake standard ≦ 14 units per week (low alcohol consumption). Therefore, in this Japanese cohort, alcohol consumers were defined as those consuming ≧ 20 g of alcohol per day, regardless of sex, in accordance with established Japanese clinical guidance. Surgical patients with alcoholic liver disease (ALD) experienced more adverse events and a higher risk of in‐hospital mortality after non‐hepatic surgeries, which was approximately 2.6 times higher than that for non‐ALD patients [25]. It was not clear whether the incidence of complications was influenced by the amount of alcohol consumed. A systematic review and meta‐analysis showed no relationship between low to moderate alcohol consumption and postoperative complications; alternatively, high alcohol consumption (> 24 g/day for women, > 36 g/day for men, Alcohol Use Disorders Test > 8, alcohol abuse, or dependency) increased the risk of postoperative mortality [27].

Conversely, in another study, alcohol consumption was not identified as an independent risk factor for SSI and anastomotic leakage when factors like age and smoking are adjusted for; moreover, no dose–response relationship was observed between alcohol consumption and SSI [28].

Although excessive alcohol consumption has been reported to impair immune system, the effects of low or moderate alcohol consumption on immune function remain unclear [26]. In this study, we found a strong association between SSI onset and alcohol consumption, suggesting that even moderate alcohol intake results in immune dysfunction and causes SSI. Patients in the alcohol consumption group were older, more frequently smokers, had lower body mass index, and exhibited higher preoperative aspartate aminotransferase and alanine aminotransferase levels (Tables S5 and S6). While none of these factors alone directly indicate immune dysfunction, their coexistence may reflect a physiological state that indirectly influences perioperative immune responses. Because immunological parameters were not directly measured in this study, the role of immune dysfunction should be interpreted as a potential indirect mechanism rather than a definitive causal explanation. The currently available literature shows a high level of clinical and moderate statistical heterogeneity, necessitating large population‐based studies to assess postoperative outcomes after gastrointestinal surgery according to low, moderate, or high alcohol intake [26].

In many cases, alcohol consumption is self‐reported, and the recommendations for alcohol intake vary from country to country [29], which could influence the occurrence of SSIs. Thus, an accurate assessment of preoperative alcohol consumption, duration of abstinence, methods of abstinence, and ways to confirm abstinence is essential.

In our previous randomized controlled trial [9], PI irrigation for surgical wounds had no additional benefit compared to irrigation with normal saline, and it increased the incidence of incisional SSI in secondary endpoints. The same results were observed in the current study, which focused solely on the MIS group. In the multivariate analysis, the use of immunosuppressive drugs was strongly related to organ/space SSI. Immunosuppressive drugs have been reported as a risk factor for SSI. However, the interpretation of the result should be approached with caution in the present study, as only a few patients were using immunosuppressive drugs (*n* = 25; 4.7%), resulting in a wide 95% confidence interval. Operation time was also associated with SSI, consistent with a previous report [15]. Although the OR of 1.003 appears small, caution is warranted with prolonged operation times, even during MIS.

Diabetes mellitus is widely recognized as a risk factor for postoperative SSI. In our cohort, diabetes was not significantly associated with SSI, which may be partly explained by intensive perioperative glycemic control. In particular, 10 of the 78 diabetic patients with poor glycemic control were admitted preoperatively for glucose management; no SSI occurred in these patients. Therefore, despite the absence of an association in this study, diabetes may be a risk factor. This observation indicates that appropriate perioperative management may mitigate SSI risk.

According to our findings, preoperative screening for alcohol consumption may help identify patients at increased risk for SSI even in the setting of MIS. Preoperative counseling and alcohol reduction or cessation programs may represent potential preventive strategies. Phosphatidylethanol is a direct biomarker of alcohol consumption within the past 1–4 weeks, making it a potential tool in the subclassification of patients with steatotic liver disease [30]. Because self‐reported alcohol intake may be underestimated, objective biomarkers such as phosphatidylethanol may be useful in future studies to improve exposure classification.

This study had several limitations. First, this study was the secondary analysis of a randomized controlled trial conducted by a single institution. Although it shows that alcohol consumption is significantly associated with SSI occurrence, this is based on post hoc and exploratory analysis. To evaluate the efficacy of preoperative alcohol abstinence in preventing SSI, a prospective study is required. In addition, minimally invasive PD was not included in this study. PD is a surgical procedure with a high incidence of SSI. However, minimally invasive PD is performed at a limited number of facilities in Japan, excluding our institution, and is not widespread. Third, alcohol consumption was self‐reported. Regarding alcohol consumption and frequency, there is a possibility of underreporting in self‐reported data. In future a prospective multicenter trial including the various procedures of MIS in gastroenterological surgery would be necessary.

## 5 Conclusion

In conclusion, alcohol consumption was independently associated with SSI in the gastroenterological MIS. It is unclear whether prohibiting alcohol consumption before surgery and the duration of consumption helps prevent the occurrence of SSI. Additional studies are warranted to explore these factors.

## Author Contributions

**Toshiya Akai:** conceptualization, methodology, writing – original draft, investigation, formal analysis, visualization. **Makoto Takeda:** conceptualization, investigation, methodology, writing – review and editing. **Eisuke Booka:** investigation, resources. **Tomohiro Matsumoto:** investigation, resources. **Masayo Takaoka:** data curation. **Mayu Sakata:** investigation, resources. **Yoshifumi Morita:** investigation, resources. **Hirotoshi Kikuchi:** investigation, resources. **Yoshihiro Hiramatsu:** investigation, resources. **Hiroya Takeuchi:** conceptualization, writing – review and editing, supervision.

## Funding

The authors have nothing to report.

## Ethics Statement

The study protocol was approved by the Clinical Research Review Board of Hamamatsu University School of Medicine (Approval No. 24‐040). The trial was designed and conducted by Hamamatsu University School of Medicine with approval from its ethics committee and in accordance with the ethical principles outlined in the Declaration of Helsinki.

## Conflicts of Interest

Hiroya Takeuchi is an editorial board member of Annals of Gastroenterological Surgery. The other authors declare no conflicts of interest.

## Supporting information


**Table S1:** Subgroup analysis according to organ system for all SSI occurrence.
**Table S2:** Comparison of the preoperative blood test results between the two groups based on SSI occurrence.
**Table S3:** Comparison of the preoperative blood test results between the two groups based on incisional SSI occurrence.
**Table S4:** Comparison of the preoperative blood test results between the two groups based on organ/space SSI occurrence.
**Table S5:** Characteristics of alcohol drinkers.
**Table S6:** Comparison of the preoperative blood test results between the two groups based on alcohol consumption.
