# Supplementary material for: Alcohol Consumption Is a Risk Factor of Surgical Site Infection After Minimally Invasive Surgery: A Secondary Observational Analysis of a Clinical Trial
Source: Ann Gastroenterol Surg. 2026 Mar 2;10(4):1337–45. doi: 10.1002/ags3.70210 (PMC13326814; doi:10.1002/ags3.70210)
Supplement: Supplementary file 1 — Table S1: Subgroup analysis according to organ system for all SSI occurrence. Table S2: Comparison of the preoperative blood test results between the two groups based on SSI occurrence. Table S3: Comparison of the preoperative blood test results between the two groups based on incisional SSI occurrence. Table S4: Comparison of the preoperative blood test results between the two groups based on organ/space SSI occurrence. Table S5: Characteristics of alcohol drinkers. Table S6: Comparison of the preoperative blood test results between the two groups based on alcohol consumption. [file AGS3-10-1337-s001.docx]

**Table S1. Subgroup analysis according to organ system for all SSI occurrence**

| Surgical site | **OR** | **95% CI** | **p-value** |
| --- | --- | --- | --- |
| Upper gastrointestinal | 1.550 | 0.908-2.648 | 0.106 |
| Lower gastrointestinal | 0.703 | 0.405-1.220 | 0.209 |
| Hepatobiliary pancreatic | 0.881 | 0.452-1.717 | 0.709 |

CI, confidence interval; OR, odds ratio; SSI, surgical site infection.

**Table S2. Comparison of the preoperative blood test results between the two groups based on SSI occurrence**

|  | **SSI (+)**  **N = 62** | **SSI (−)**  **N = 467** | **p-value** |
| --- | --- | --- | --- |
| White blood cells (/μL) | 5545 (4302–6407) | 5610 (4680–6760) | 0.262 |
| Hemoglobin (g/dL) | 13.2 ± 1.9 | 12.9 ± 1.7 | 0.268 |
| Platelets (×10^4^/μL) | 23.1 (19.0–28.7) | 24.4 (20.4–29.6) | 0.208 |
| Total bilirubin (mg/dL) | 0.70 (0.50–0.80) | 0.60 (0.50–0.80) | 0.355 |
| Aspartate aminotransferase (U/L) | 21.5 (18.0–26.0) | 20.0 (17.0–25.0) | 0.204 |
| Alanine aminotransferase (U/L) | 17.0 (12.7–26.0) | 17.0 (13.0–24.0) | 0.698 |
| Albumin (g/dL) | 4.1 (3.7–4.4) | 4.2 (3.9–4.4) | 0.138 |
| Blood urea nitrogen (mg/dL) | 15.4 (12.8–17.7) | 14.9 (12.1–18.6) | 0.386 |
| Creatinine (mg/dL) | 0.83 (0.71–0.97) | 0.78 (0.64–0.93) | 0.034 |
| C-reactive protein (mg/dL) | 0.11 (0.05–0.30) | 0.11 (0.05–0.25) | 0.514 |
| Prothrombin time (%) | 106.0 (95.7–114.5) | 107.0 (98.0–118.0) | 0.572 |
| Activated partial thromboplastin time (%) | 82.0 (64.7–107.7) | 86.0 (71.0–108.5) | 0.268 |

Continuous variables are expressed as median (interquartile range) due to non-normal distribution and as mean ± standard deviation due to normal distribution

**Table S3. Comparison of the preoperative blood test results between the two groups based on incisional SSI occurrence**

|  | **SSI (+)**  **N = 29** | **SSI (−)**  **N = 500** | **p-value** |
| --- | --- | --- | --- |
| White blood cells (/μL) | 5750 (4245–6570) | 5585 (4660–6750) | 0.818 |
| Hemoglobin (g/dL) | 13.3 ± 1.9 | 12.9 ± 1.7 | 0.200 |
| Platelets (×10^4^/μL) | 24.3 (21.5–28.8) | 24.2 (19.7–29.5) | 0.983 |
| Total bilirubin (mg/dL) | 0.70 (0.59–0.87) | 0.60 (0.50–0.80) | 0.220 |
| Aspartate aminotransferase (U/L) | 24.0 (19.0–32.5) | 20.0 (17.0–25.0) | 0.016 |
| Alanine aminotransferase (U/L) | 20.0 (12.5–34.0) | 17.0 (13.0–24.0) | 0.139 |
| Albumin (g/dL) | 4.0 (3.6–4.4) | 4.2 (3.9–4.4) | 0.227 |
| Blood urea nitrogen (mg/dL) | 14.2 (12.3–17.6) | 14.9 (12.2–18.6) | 0.903 |
| Creatinine (mg/dL) | 0.78 (0.70–0.91) | 0.78 (0.65–0.94) | 0.977 |
| C-reactive protein (mg/dL) | 0.14 (0.06–0.34) | 0.11 (0.05–0.25) | 0.297 |
| Prothrombin time (%) | 107.0 (94.5–120.0) | 107.0 (97.0–117.0) | 0.856 |
| Activated partial thromboplastin time (%) | 85.0 (65.5–118.5) | 85.0 (70.0–107.0) | 0.973 |

Continuous variables are expressed as median (interquartile range) due to non-normal distribution and as mean ± standard deviation due to normal distribution

**Table S4. Comparison of the preoperative blood test results between the two groups based on organ/space SSI occurrence**

|  | **SSI (+)**  **N = 33** | **SSI (−)**  **N = 496** | **p-value** |
| --- | --- | --- | --- |
| White blood cells (/μL) | 4970 (4175–6290) | 5610 (4652–6750) | 0.202 |
| Hemoglobin (g/dL) | 13.0 ± 1.9 | 12.9 ± 1.7 | 0.790 |
| Platelets (×10^4^/μL) | 20.5 (17.5–28.4) | 24.4 (20.5–29.5) | 0.090 |
| Total bilirubin (mg/dL) | 0.61 (0.50–0.90) | 0.61 (0.50–0.80) | 0.941 |
| Aspartate aminotransferase (U/L) | 20.0 (18.0–23.5) | 20.0 (17.0–26.0) | 0.555 |
| Alanine aminotransferase (U/L) | 15.0 (12.5–24.5) | 17.0 (13.0–25.0) | 0.381 |
| Albumin (g/dL) | 4.1 (3.7–4.4) | 4.2 (3.9–4.4) | 0.403 |
| Blood urea nitrogen (mg/dL) | 15.4 (13.0–19.6) | 14.8 (12.1–18.5) | 0.205 |
| Creatinine (mg/dL) | 0.87 (0.73–1.05) | 0.78 (0.65–0.92) | 0.005 |
| C-reactive protein (mg/dL) | 0.10 (0.05–0.27) | 0.11 (0.05–0.25) | 0.910 |
| Prothrombin time (%) | 106.0 (96.0–112.0) | 107.0 (97.7–118.0) | 0.356 |
| Activated partial thromboplastin time (%) | 81.0 (63.5–98.0) | 86.0 (70.0–109.0) | 0.149 |

Continuous variables are expressed as median (interquartile range) due to non-normal distribution and as mean ± standard deviation due to normal distribution

**Table S5. Characteristics of alcohol drinkers**

|  | **Alcohol consumption (+)**  **N = 189** | **Alcohol consumption (−)**  **N = 340** | **p-value** |
| --- | --- | --- | --- |
| Sex: male/female | 162 (%)/27 (%) | 158 (%)/182 (%) | <0.001 |
| Age (years), median (IQR) | 70.0 (61.0–77.0) | 65.0 (51.2–74.0) | <0.001 |
| Current smoking | 54 (%) | 45 (%) | <0.001 |
| ASA-PS: Ⅰ, Ⅱ/Ⅲ | 169 (%)/20 (%) | 41 (%)/299 (%) | 0.363 |
| BMI (kg/m^2^), median (IQR) | 22.1 (20.3–24.5) | 23.0 (20.5–26.4) | 0.008 |
| Diabetes | 24 (%) | 54 (%) | 0.322 |
| Immunosuppressive drugs | 7 (%) | 18 (4.0%) | 0.409 |

ASA-PS, American Society of Anesthesiologists–Physical Status; BMI, body mass index; SSI, surgical site infection.

**Table S6. Comparison of the preoperative blood test results between the two groups based on alcohol consumption**

|  | **Alcohol consumption (+)**  **N = 189** | **Alcohol consumption (−)**  **N = 340** | **p-value** |
| --- | --- | --- | --- |
| White blood cells (/μL) | 5560 (4590–6500) | 5610 (4660–6887) | 0.181 |
| Hemoglobin (g/dL) | 13.0 ± 1.8 | 12.9 ± 1.7 | 0.310 |
| Platelets (×10^4^/μL) | 23.8 (19.1–29.2) | 24.6 (20.9–29.8) | 0.106 |
| Total bilirubin (mg/dL) | 0.66 (0.50–0.80) | 0.60 (0.50–0.80) | 0.184 |
| Aspartate aminotransferase (U/L) | 22.0 (19.0–28.5) | 19.5 (17.0–24.0) | <0.001 |
| Alanine aminotransferase (U/L) | 19.0 (13.0–27.0) | 16.0 (13.0–23.0) | 0.013 |
| Albumin (g/dL) | 4.1 (3.9–4.4) | 4.2 (3.9–4.4) | 0.176 |
| Blood urea nitrogen (mg/dL) | 15.5 (12.8–19.0) | 14.6 (11.7–18.3) | 0.018 |
| Creatinine (mg/dL) | 0.84 (0.73–0.98) | 0.74 (0.62–0.91) | <0.001 |
| C-reactive protein (mg/dL) | 0.12(0.06–0.30) | 0.10(0.05–0.23) | 0.077 |
| Prothrombin time (%) | 106.0 (97.0–118.0) | 107.0 (98.0–117.2) | 0.982 |
| Activated partial thromboplastin time (%) | 87.0 (71.0–113.5) | 84.0 (69.0–107.0) | 0.193 |

Continuous variables are expressed as median (interquartile range) due to non-normal distribution and as mean ± standard deviation due to normal distribution
